# Crystal structure of bis­(2-bromo­ethyl­ammonium) hexa­bromido­stannate(IV)

**DOI:** 10.1107/S2056989025010588

**Published:** 2026-01-01

**Authors:** Danylo S. Kreiman, Dmytro M. Korytko, Iryna S. Kuzevanova, Mihaela Dascalu, Il’ya A. Gural’skiy

**Affiliations:** a Department of Chemistry, Taras Shevchenko National University of Kyiv, Volodymyrska st. 64/13, 01601 Kyiv, Ukraine; b "Petru Poni" Institute of Macromolecular Chemistry Romanian Academy of Science Aleea Grigore Ghica Voda 41-A 700487 Iasi Romania; Vienna University of Technology, Austria

**Keywords:** crystal structure, bis­(2-bromo­ethanamine) hexa­bromido­stannate(IV), tin(IV) bromide, metal halides

## Abstract

In the hybride title salt, (C_2_H_7_BrN)_2_[SnBr_6_], the charge of the anionic [SnBr_6_]^2−^ moiety is balanced by two (H_3_N(CH_2_)_2_Br)^+^ cations. The tin(IV) atom is located on a mirror plane and has a slightly distorted octa­hedral coordination environment. The inorganic octa­hedra are discrete, thus leading to a 0D topology within the crystal structure. The two crystallographically unique organic cations have different conformations: while one has a *gauche* conformation, the other has an *anti* conformation, both without special symmetry but with positional disorder over the crystallographic mirror plane. Contacts between organic and inorganic parts in the crystal structure are ensured by N—H⋯Br hydrogen bonds and weak Br⋯Br and C—H⋯Br inter­actions.

## Abstract 2

Bis(2-bromo­ethanamminium) hexa­bromido­stannate(IV) is a hybrid tin perovskite with 0D topology due to the presence of isolated octa­hedral [SnBr_6_]^2–^ anions.

## 1. Chemical context

Hybrid metal halides with perovskite-type structures are an important class of solution-processed semiconductors with noteworthy electronic and optical behavior. The most studied are Pb-based perovskites, but inclusion of toxic lead makes the resulting product rather inapplicable. To reduce the toxicity of the resulting perovskites, Pb is frequently replaced with less toxic elements like Sn, Ge, Cu, Sb, or Bi. Sn-based hybrid perovskites were found to be the most promising ones in terms of their optoelectric properties (Wang & Shi, 2024 ▸). Notably, during storage in air, tin can oxidize from Sn^II^ to Sn^IV^, which is usually a drawback, but Sn^IV^-based materials have still found some important applications. For example, Sn^IV^ can play beneficial roles in perovskites when deliberately engineered at surfaces or in the bulk of oxide-based materials. In inorganic CsPb_0.6_Sn_0.4_I_3_, sequential surface Sn^IV^ hydrolysis leads to an ultrathin *n*-type tin-oxide layer that passivates traps and optimizes band alignment, raising power conversion efficiency to 16.79% with T90 ≃ 958 h, illustrating purposeful the use of Sn^IV^ as an inter­facial component rather than a defect (Hu *et al.*, 2023 ▸). Deliberately maintained oxidized tin at surfaces or grain boundaries can also assist passivation, barrier formation, and contact selectivity in tin perovskite optoelectronics (Yang *et al.*, 2025 ▸).

**Figure 1:**
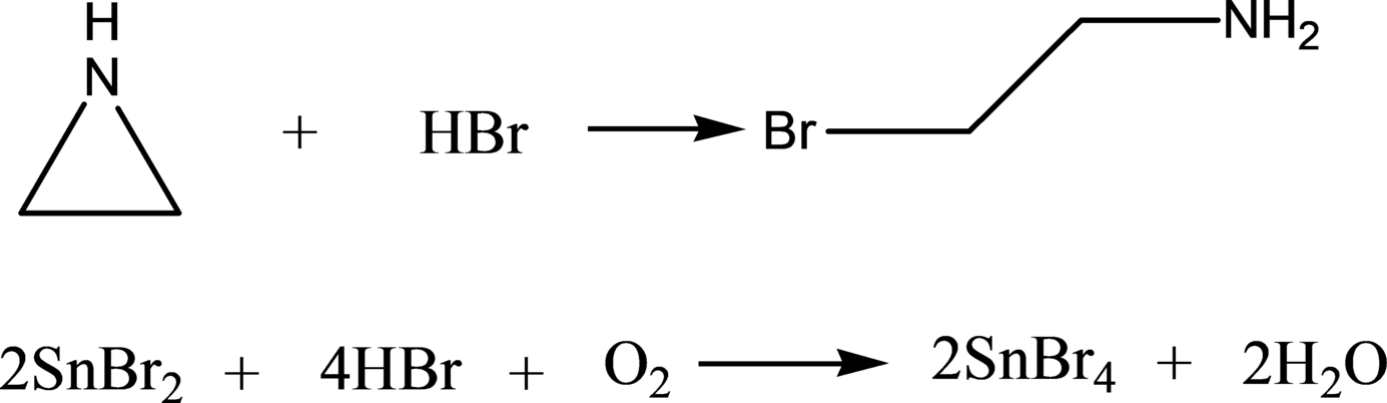
Reaction scheme for ring opening of aziridine, and of the oxidation of Sn^IV^ to Sn^IV^.

Apart from well-studied hybrid perovskites with methyl­ammonium and formamidinium cations, materials containing the aziridinium cation have gained attention in the past few years. The small size of the aziridinium cation suits the perovskite tolerance window (Teng *et al.*, 2021 ▸) and promotes stabilization of 3D halide frameworks (Petrosova *et al.*, 2022 ▸). Combining the reduced toxicity of tin with the small aziridinium ring cation, (AzrH)SnHal_3_ (Hal is a halogen) perovskites can stabilize 3D frameworks and maintain semiconducting properties for multiple halides, positioning them as attractive lead-free materials for light absorption and emission (Kucheriv *et al.*, 2023 ▸). At the same time, working with aziridinium tin halide perovskite requires additional caution due to the tendency of tin(II) to oxidize to tin(IV) and of aziridinium to undergo ring opening (Fig. 1 ▸).

In this work we report on the crystal structure of bis­(2-bromo­ethyl­ammonium) hexa­bromido­stanate(IV), which has formed unintentionally upon the intended synthesis of (AzrH)SnBr_3_.
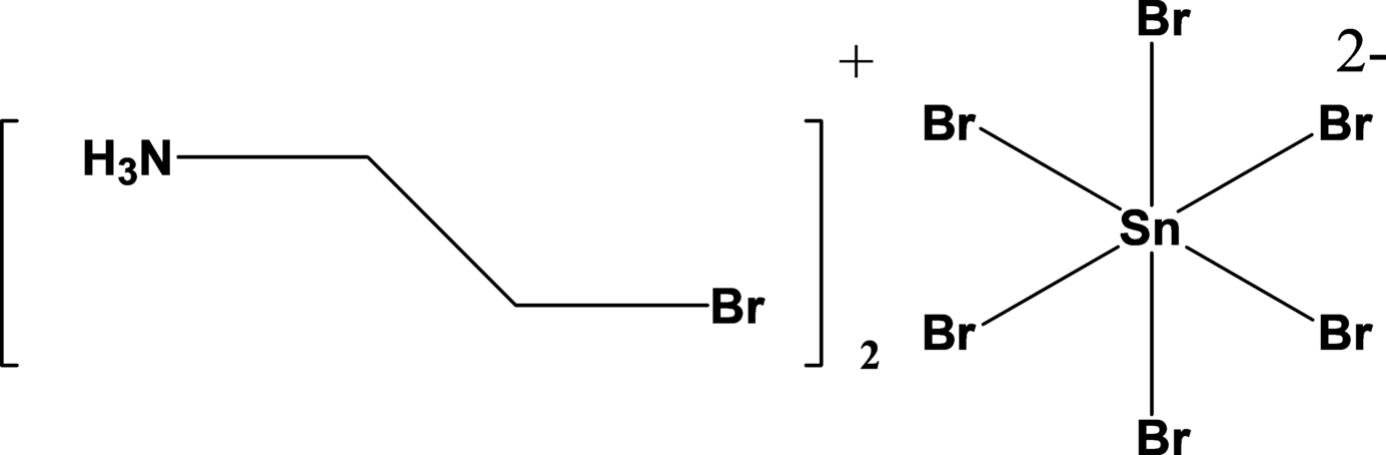


## 2. Structural commentary

**Figure 2:**
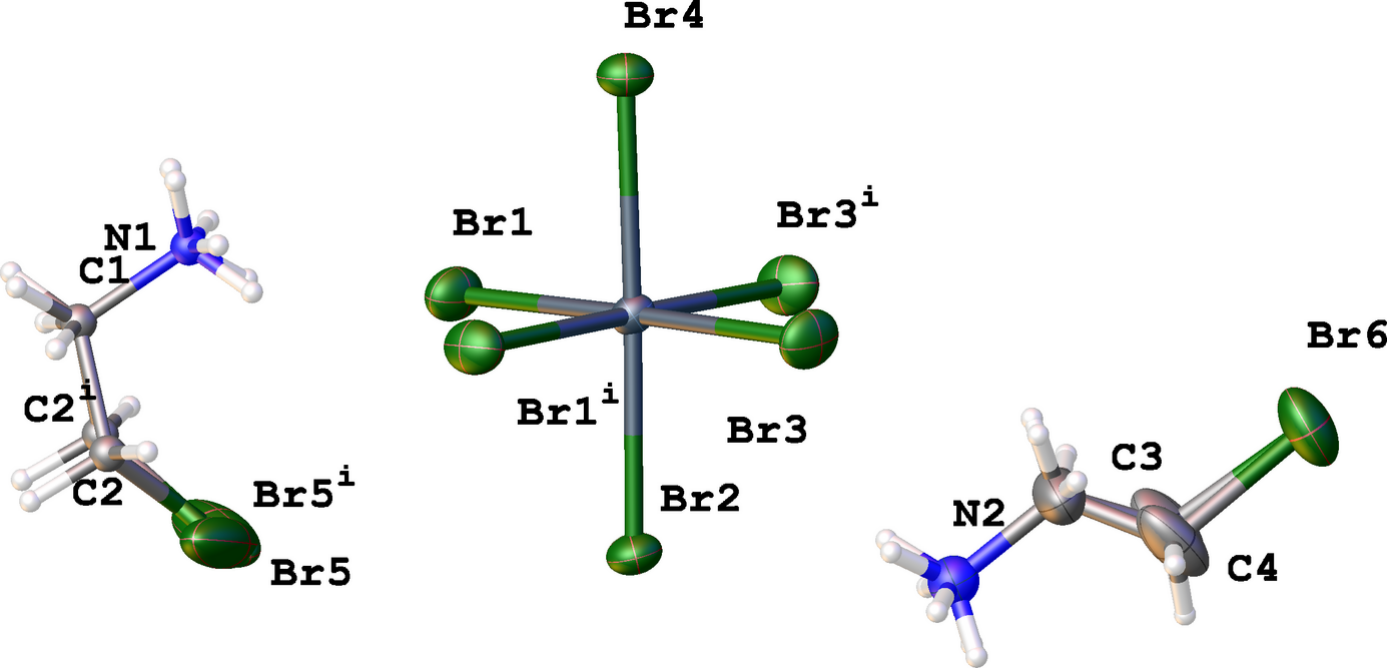
The building units in the crystal structure of the title compound, showing the atom-labelling scheme [symmetry code: (i) *x*, 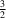 − *y*, *z*]. Displacement ellipsoids are drawn at the 50% probability level; disorder of the two cations is shown.

The crystal structure of the title compound consists of two organic 2-bromo­ethyl­ammonium cations and octa­hedral [SnBr_6_]^2–^ anions (Fig. 2 ▸). The backbone of the cation N1—C1—C2—Br5 has a torsional angle of −65.4 (13)° and thus adopts a *gauche* conformation, while that of the other cation N2—C3—C4—Br6 has a torsional angle of 165.9 (7)° and adopts an *anti* conformation. Both cations are equally disordered over a mirror plane. This disorder affects atoms C2 and Br5 of the first cation and C4 of the second cation, as well as the H atoms bonded to N1, N2, C1, and C3. The neighbouring [SnBr_6_]^2–^ octa­hedra do not inter­act directly with each other, leading to a 0D topology within the crystal structure. The Sn—Br bond lengths vary from 2.5662 (10) to 2.6135 (16) Å. The [SnBr_6_]^2–^ octa­hedron is distorted with notable elongation of axial bonds: Sn1—Br4 and Sn1—Br2 bond length are 2.6127 (16) Å and 2.6135 (16) Å, respectively, while the bond lengths Sn1—Br1 and Sn1—Br3 (and two symmetry equivalents generated by a mirror plane) with ligands in equatorial positions are 2.5828 (10) Å and 2.5662 (10) Å, respectively. The angles Br4—Sn1—Br2 and Br3—Sn1—Br1 are almost equal, 178.52 (5) and 178.72 (4)°, with minimal deviation from the ideal 180°. The *cis*-Br—Sn—Br angles vary from 89.11 (4) to 91.82 (5)°, which also shows a very small deviation from 90°. Qu­anti­tative octa­hedral distortion parameters were calculated as Δ*d* = (1/6)Σ^6^_*i*=1_(*d*_i_ − *d*)^2^/*d*^2^ (1) and Σ=^12^_*i*=1_|90 − α_*i*_| (2) where *d_i_* is the Sb—Br bond length and d is the average bond length, and α_*i*_ corresponds to 12 *cis*-angles in the octa­hedron. The value of Δ*d* is 5.64 × 10^−5^, which is typical for a perovskite structure with 0D topology. The Σ value amounts to 8.669°.

## 3. Supra­molecular features

**Figure 3:**
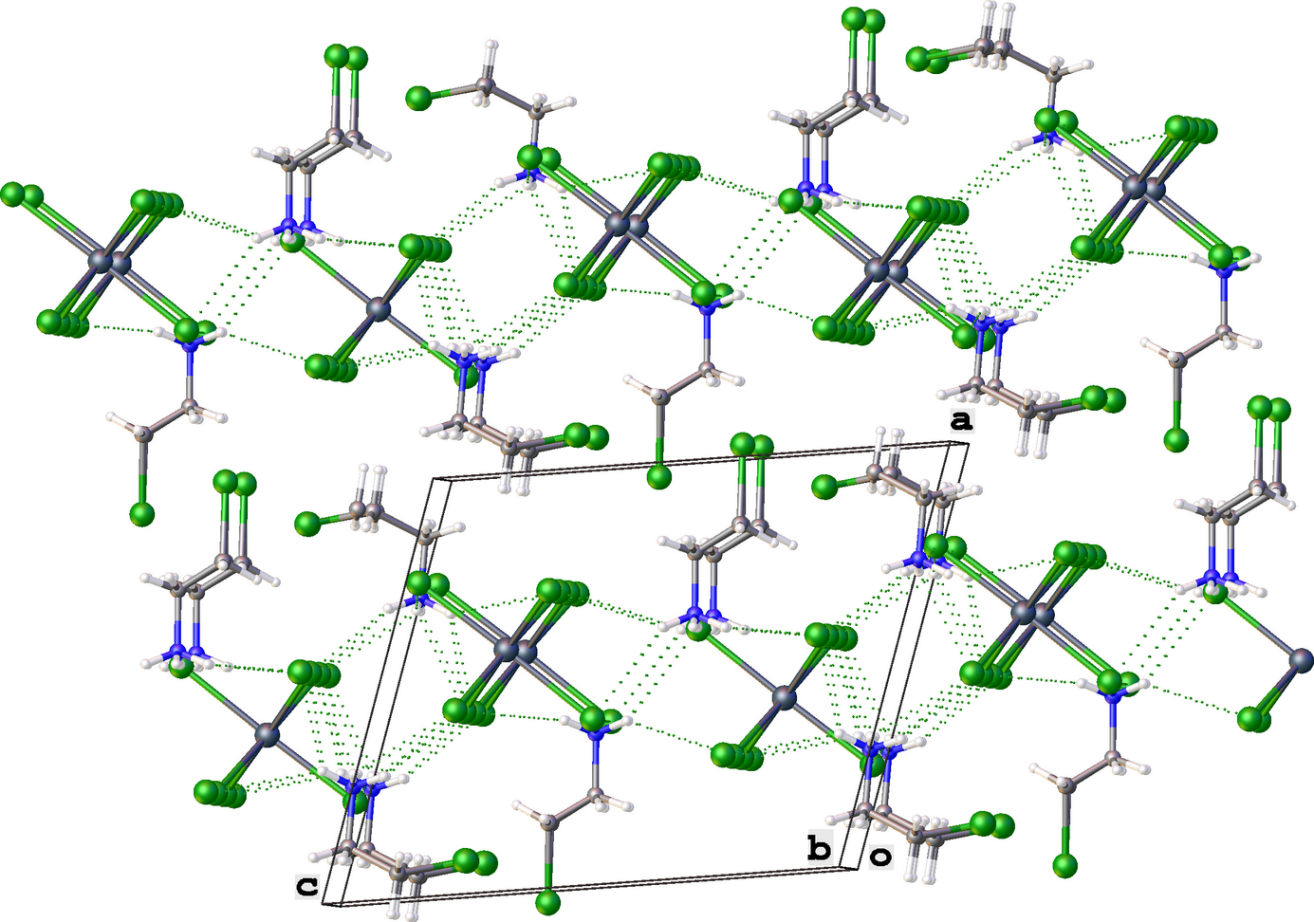
N—H⋯Br hydrogen bonds between cations and anions create supra­molecular layers. The second part of disordered organic cations was omitted for clarity.

Fig. 3 ▸ shows a fragment of the crystal structure and illustrates the inter­molecular organization through N—H⋯Br hydrogen bonds, formed between ammonium groups and the Br atoms of the [SnBr_6_]^2–^ anions, which create supra­molecular layers parallel to the *bc* plane (Figs. 3 ▸, 4 ▸). The strongest hydrogen bonds are N2—H2D⋯Br2 and N1—H1*A*⋯Br1(−*x* + 1, −*y* + 2, −*z*), with *D*⋯*A* distances of 3.462 (10) and 3.481 (9) Å, and N—H⋯Br angles of 133 and 134°, respectively. Numerical data of other N—H⋯Br inter­actions are given in Table 1 ▸. Notably, N—H⋯Br hydrogen bonds are not realized between the ammonium group and the Br atoms of neighbouring cations. Instead, a close Br2⋯Br6(1 + *x*, *y*, *z*) contact [3.704 (2) Å] is observed between the bromine atom of an organic cation and one of the bromido ligands [C4—Br6(1 + *x*, −*y*, *z*)⋯Br2 = 159.3689 (11)°, Sn1—Br6(1 + *x*, −*y*, *z*)⋯Br2 = 143.209 (3)°]. The arrangement of this Br⋯Br inter­action suggests partial σ-hole directionality, although the distance is at the van der Waals limit (3.7 Å for Br⋯Br; Bondi, 1964 ▸), indicating a weak halogen-type inter­action rather than a strong halogen bond.

**Figure 5:**
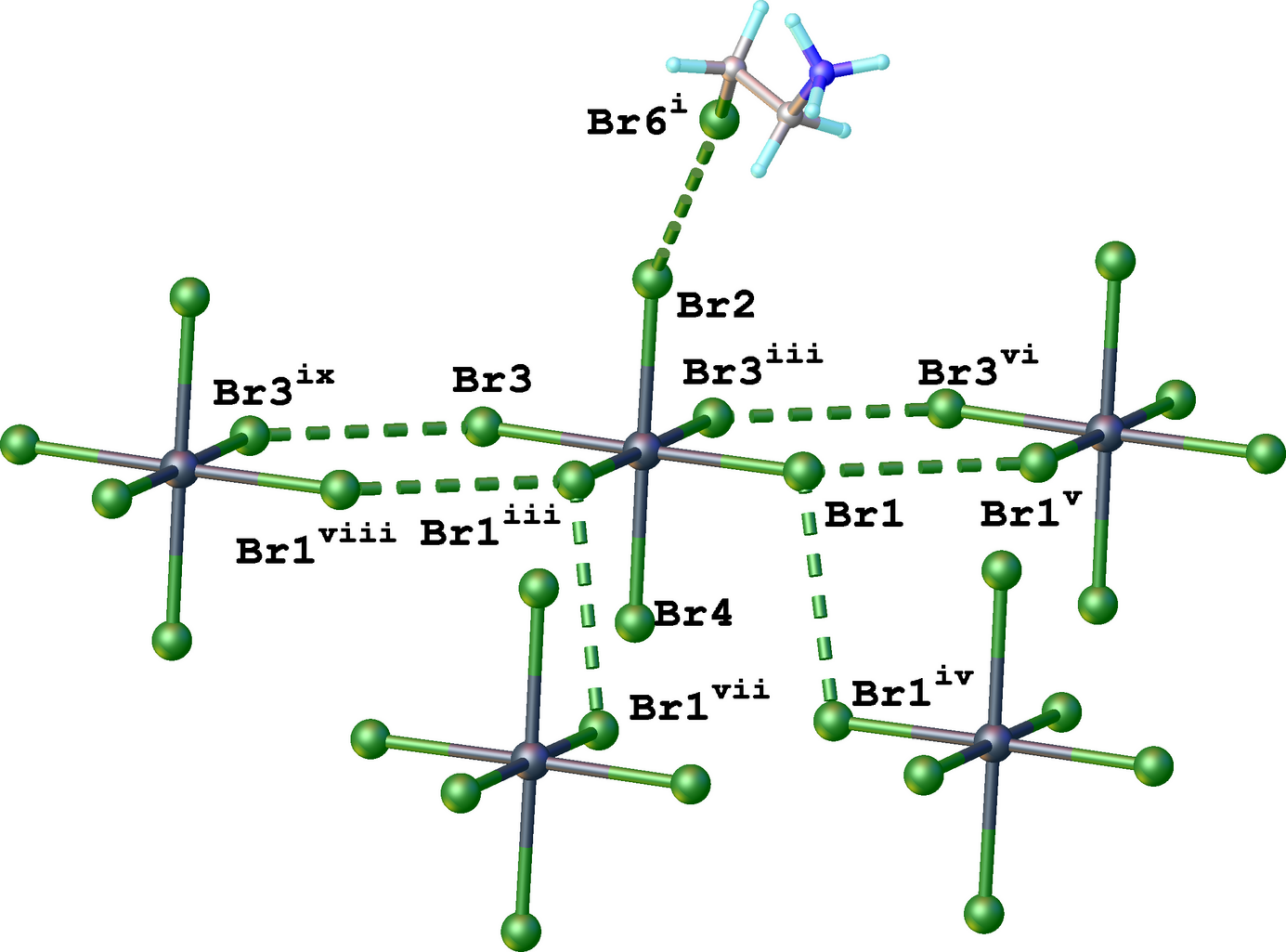
View of a fragment of the crystal structure of bis­(2-bromo­ethyl­ammonium) hexa­bromido­stannate(IV) showing the Br⋯Br contacts as green dashed lines [symmetry codes: (i) 1 + *x*, +*y*, +*z*; (iii) *x*, 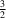 − *y*, +*z*; (iv) −*x* + 1, 2 − *y*, −*z*; (v) +*x*, 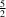 − *y*, +*z*; (vi) +*x*, 1 + *y*, +*z*; (vii) 1 − *x*, −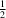 + *y*, −*z*; (viii) +*x*, −1 + *y*, +*z*; (ix) +*x*, 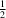 − *y*, +*z*]. The disorder of organic cations was omitted for clarity.

**Figure 6:**
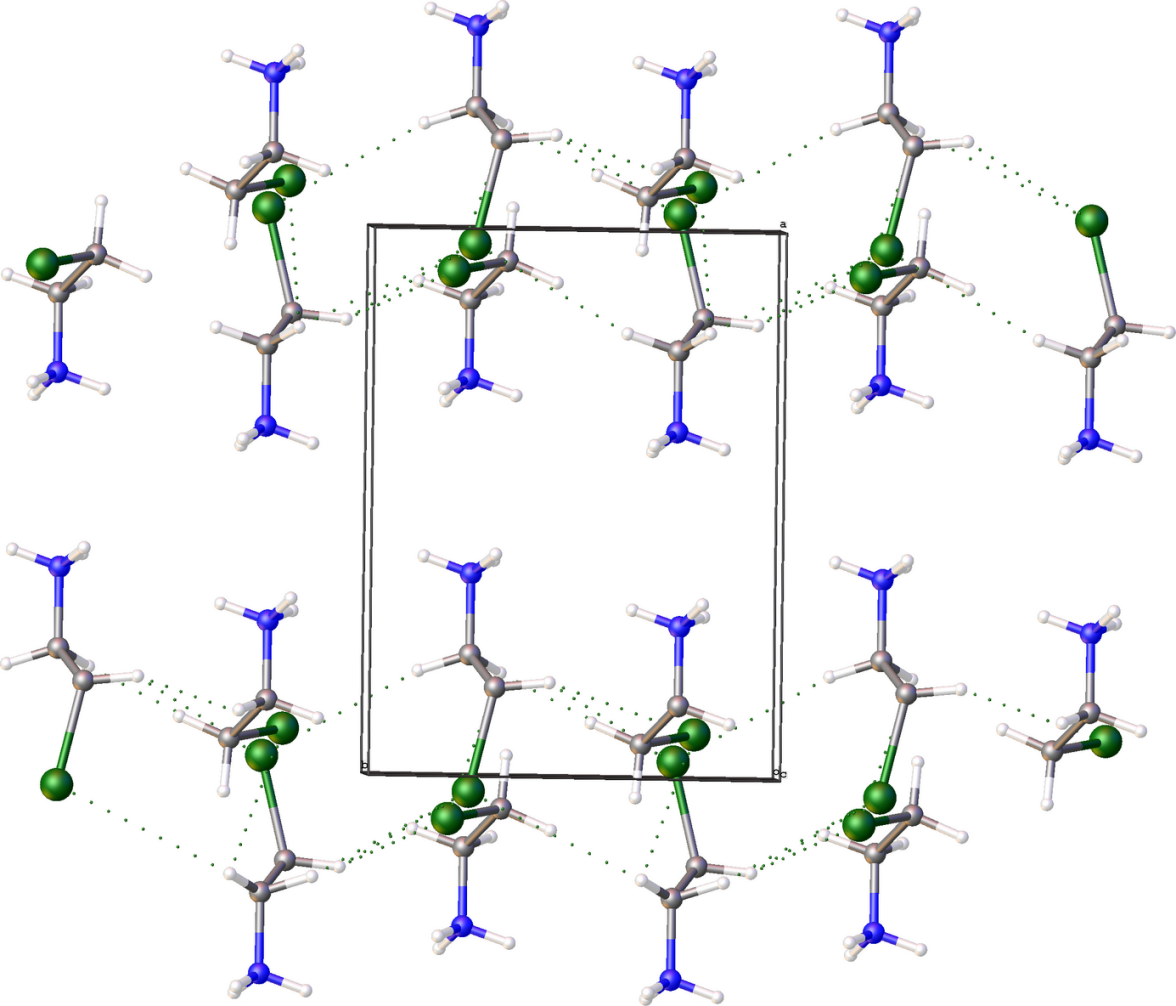
Arrangement of organic cations into supra­molecular chains through C—H⋯Br inter­actions. The cation disorder was omitted for clarity.

There are other short Br⋯Br inter­actions between neighbouring [SnBr_6_]^2–^ octa­hedra (Fig. 5 ▸) within a range of 3.7–3.8 Å, and Θ_1_ ≃ Θ_2_ (135 and 107°, accordingly). This distance corresponds to approximately the sum of van der Waals radii and can therefore inter­preted as a type I geometry-based contact (Veluthaparambath *et al.*, 2023 ▸) arising from close-packing requirements rather than a true halogen⋯halogen inter­action (Desiraju & Parthasarathy, 1989 ▸; Veluthaparambath *et al.*, 2023 ▸). This Br⋯Br contact ensures that [SnBr_6_]^2–^ octa­hedra arrange themselves into supra­molecular layers. Organic cations also arrange themselves, then into supra­molecular chains propagating parallel to the *b* axis through weak C—H⋯Br inter­actions (Fig. 6 ▸, Table 1 ▸). Additional C—H⋯Br contacts (Table 1 ▸) between the organic cations and the [SnBr_6_]^2–^ octa­hedra consolidate the packing.

## 4. Database survey

A search of the Cambridge Structure Database (CSD, version 6.00, last update April 2025; Groom *et al.*, 2016 ▸) revealed 102 structures containing the 2-bromo­ethyl­ammonium cation of the title compound. Selected examples include OJAPIC (Luo *et al.*, 2023 ▸) and NUSRIF (Ishihara *et al.*, 2020 ▸). OJAPIC is (2-bromo­etyl­ammonium)_3_[InBr_6_] containing discrete [InBr_6_] octa­hedra with an 0D topology similar to that of the title compound. NUSRIF is (2-bromo­etyl­ammonium)_2_[CdBr_4_] and is made up from [CdBr_6_] octa­hedra connected into layers through corner-sharing.

## 5. Synthesis and crystallization

Tin(II) chloride (150 mg, 0.79 mmol, 1 eq.) was dissolved in 1 ml of water and 0.1 ml of HCl (to avoid hydrolysis). Ammonia solution (0.5 ml) was added to the first solution and stirred. As a result, a white precipitate of Sn(OH)_2_ was formed. The precipitate was filtered off and washed with water. The obtained tin hydroxide was dissolved in a mixture of 2.4 ml of hydro­bromic acid (48%_wt_) and 4.5 ml of water. Aziridine (120 µl, 2.3 mmol, 2.9 eq.) were dissolved in 2 ml of water, previously cooled in an ice bath. The aziridine solution was then added dropwise to tin bromide solution in an ice bath under stirring (Kucheriv *et al.*, 2023 ▸). After that, the solution was left in the air for a month to produce crystals of the title compound.

## 6. Refinement

Crystal data, data collection and structure refinement details are summarized in Table 2 ▸. Disorder is caused by a mirror plane parallel to the *ac* plane. Occupancies of C2, C4 and Br5 were set to 0.5. Hydrogen atoms bonded to C1, C3, N1, N2 are also disordered over this mirror plane. One hydrogen atom (H4*A*) was considered to be part of two disordered moieties [C4, C4(*x*, 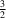 − *y*, *z*)]. H atoms were placed at calculated positions and refined with *U*_iso_(H) = 1.2*U*_eq_(C) or *U*_iso_(H) = 1.2*U*_eq_(N). H atoms of CH_2_ groups were refined as riding and of NH_3_ groups as rotating.

## Supplementary Material

Crystal structure: contains datablock(s) I. DOI: 10.1107/S2056989025010588/wm5776sup1.cif

Structure factors: contains datablock(s) I. DOI: 10.1107/S2056989025010588/wm5776Isup2.hkl

CCDC reference: 2505907

Additional supporting information:  crystallographic information; 3D view; checkCIF report

## Figures and Tables

**Figure 4:**
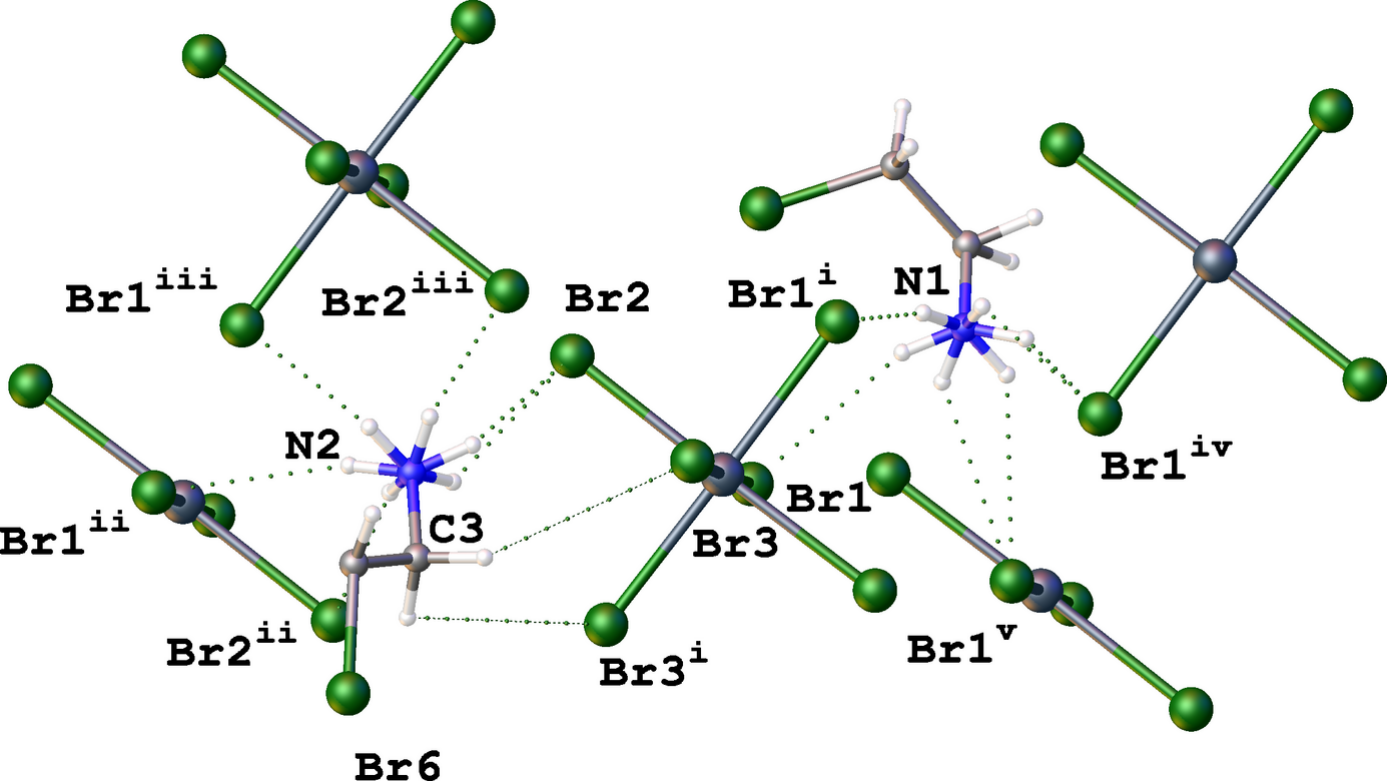
View of a fragment of the crystal structure of bis­(2-bromo­ethyl­ammonium) hexa­bromido­stannate(IV) showing the conformation of two types of organic cations, the hydrogen-bonding scheme and C—H⋯Br contacts (dotted lines) [symmetry codes: (i) −*x* + 1, −*y* + 2, −*z* + 1; (ii) −*x* + 1, *y* − 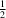, −*z* + 1; (iii) *x*, −*y* + 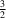, *z*; (iv) −*x* + 1, *y* − 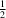, −*z*; (v) −*x* + 1, −*y* + 2, −*z*]. The disorder of organic cations, except for the H atoms of the NH_3_ groups, was omitted for clarity.

**Table 1:** Hydrogen-bond geometry (Å, °)

*D*—H⋯*A*	*D*—H	H⋯*A*	*D*⋯*A*	*D*—H⋯*A*
N1—H1*A*⋯Br1^ii^	0.90	2.79	3.481 (9)	134
N1—H1*A*⋯Br3^iii^	0.90	3.08	3.864 (11)	147
N1—H1*B*⋯Br1	0.90	2.73	3.587 (10)	160
N1—H1*C*⋯Br1^i^	0.90	3.05	3.587 (10)	121
N1—H1*C*⋯Br1^iv^	0.90	2.87	3.481 (9)	126
N1—H1*C*⋯Br4^iv^	0.90	2.94	3.7320 (11)	147
N2—H2*C*⋯Br2^v^	0.90	2.87	3.7282 (9)	161
N2—H2*D*⋯Br2	0.90	2.78	3.462 (10)	133
N2—H2*D*⋯Br3	0.90	3.09	3.861 (10)	145
N2—H2*E*⋯Br1^vi^	0.90	2.71	3.586 (9)	164
C1—H1*D*⋯Br4^iii^	0.98	3.13	3.872 (3)	133
C1—H1*E*⋯Br3^vii^	0.98	3.20	3.971 (13)	137
C2—H2*A*⋯Br3^viii^	0.98	2.91	3.73 (2)	142
C2—H2*A*⋯Br4^viii^	0.98	3.10	3.92 (2)	142
C3—H3*B*⋯Br3	0.98	3.13	3.934 (14)	140
C4—H4*B*⋯Br5^vi^	0.98	2.79	3.54 (2)	134
C4—H4*B*⋯Br6^ix^	0.98	3.42	4.08 (2)	126

Symmetry codes: (i) 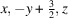; (ii) 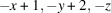; (iii) 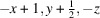; (iv) 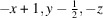; (v) 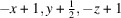; (vi) 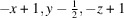; (vii) 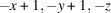; (viii) 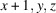; (ix) 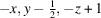.

**Table 2:** Experimental details

Crystal data	
Chemical formula	(C_2_H_7_BrN)_2_[SnBr_6_]
*M*_r_	848.14
Crystal system, space group	Monoclinic, *P*12_1_/*m*1
Temperature (K)	206
*a*, *b*, *c* (Å)	10.4048 (4), 7.4254 (3), 12.2850 (5)
β (°)	108.740 (4)
*V* (Å^3^)	898.82 (7)
*Z*	2
Radiation type	Mo *K*α
μ (mm^−1^)	19.18
Crystal size (mm)	0.3 × 0.2 × 0.03

Data collection	
Diffractometer	Xcalibur, Eos
Absorption correction	Analytical [*CrysAlis PRO* (Rigaku OD, 2024 ▸) using a multifaceted crystal model based on expressions derived by Clark & Reid (1995 ▸)]
*T*_min_, *T*_max_	0.047, 0.589
No. of measured, independent and observed [*I* > 2σ(*I*)] reflections	6106, 2293, 1492
*R*_int_	0.052
(sin θ/λ)_max_ (Å^−1^)	0.687

Refinement	
*R*[*F*^2^ > 2σ(*F*^2^)], *wR*(*F*^2^), *S*	0.051, 0.118, 1.08
No. of reflections	2293
No. of parameters	96
H-atom treatment	H-atom parameters constrained
Δρ_max_, Δρ_min_ (e Å^−3^)	1.21, −1.22

Computer programs: *CrysAlis PRO* (Rigaku OD, 2024 ▸), *SHELXT* (Sheldrick, 2015*a* ▸), *SHELXL* (Sheldrick, 2015*b* ▸), *OLEX2* (Dolomanov *et al.*, 2009 ▸) and *publCIF* (Westrip, 2010 ▸).

## supplementary crystallographic information

### Bis(2-bromoethylammonium) hexabromidostannate(IV) Crystal data

**Table:**

(C_2_H_7_BrN)_2_[SnBr_6_]	*F*(000) = 764
*M**_r_* = 848.14	*D*_x_ = 3.134 Mg m^−^^3^
Monoclinic, *P*12_1_/*m*1	Mo *K*α radiation, λ = 0.71073 Å
*a* = 10.4048 (4) Å	Cell parameters from 1850 reflections
*b* = 7.4254 (3) Å	θ = 2.1–27.7°
*c* = 12.2850 (5) Å	µ = 19.18 mm^−^^1^
β = 108.740 (4)°	*T* = 206 K
*V* = 898.82 (7) Å^3^	Prism, clear intense colourless
*Z* = 2	0.3 × 0.2 × 0.03 mm

### Bis(2-bromoethylammonium) hexabromidostannate(IV) Data collection

**Table:**

Xcalibur, Eos diffractometer	2293 independent reflections
Radiation source: fine-focus sealed X-ray tube, Enhance (Mo) X-ray Source	1492 reflections with *I* > 2σ(*I*)
Graphite monochromator	*R*_int_ = 0.052
Detector resolution: 16.1593 pixels mm^-1^	θ_max_ = 29.2°, θ_min_ = 1.8°
ω scans	*h* = −12→13
Absorption correction: analytical [CrysAlisPro (Rigaku OD, 2024) using a multifaceted crystal model based on expressions derived by Clark & Reid (1995)]	*k* = −9→8
*T*_min_ = 0.047, *T*_max_ = 0.589	*l* = −15→16
6106 measured reflections	

### Bis(2-bromoethylammonium) hexabromidostannate(IV) Refinement

**Table:**

Refinement on *F*^2^	Primary atom site location: dual
Least-squares matrix: full	Hydrogen site location: mixed
*R*[*F*^2^ > 2σ(*F*^2^)] = 0.051	H-atom parameters constrained
*wR*(*F*^2^) = 0.118	*w* = 1/[σ^2^(*F*_o_^2^) + (0.0345*P*)^2^ + 1.9061*P*] where *P* = (*F*_o_^2^ + 2*F*_c_^2^)/3
*S* = 1.08	(Δ/σ)_max_ < 0.001
2293 reflections	Δρ_max_ = 1.21 e Å^−^^3^
96 parameters	Δρ_min_ = −1.22 e Å^−^^3^
0 restraints	

### Bis(2-bromoethylammonium) hexabromidostannate(IV) Special details

**Table:**

Geometry. All esds (except the esd in the dihedral angle between two l.s. planes) are estimated using the full covariance matrix. The cell esds are taken into account individually in the estimation of esds in distances, angles and torsion angles; correlations between esds in cell parameters are only used when they are defined by crystal symmetry. An approximate (isotropic) treatment of cell esds is used for estimating esds involving l.s. planes.

### Bis(2-bromoethylammonium) hexabromidostannate(IV) Fractional atomic coordinates and isotropic or equivalent isotropic displacement parameters (Å^2^)

**Table:**

	*x*	*y*	*z*	*U*_iso_*/*U*_eq_	Occ. (<1)
Sn1	0.42076 (8)	0.750000	0.22307 (7)	0.0214 (2)	
Br1	0.55926 (10)	0.99642 (13)	0.16238 (9)	0.0416 (3)	
Br2	0.60093 (13)	0.750000	0.42822 (11)	0.0346 (3)	
Br3	0.28811 (10)	0.50178 (12)	0.28600 (9)	0.0424 (3)	
Br4	0.24572 (12)	0.750000	0.01568 (11)	0.0327 (3)	
Br5	0.9188 (2)	0.7938 (10)	0.2520 (2)	0.113 (3)	0.5
Br6	−0.02932 (16)	0.750000	0.58338 (18)	0.0678 (6)	
N1	0.7216 (10)	0.750000	−0.0082 (10)	0.043 (3)	
H1A	0.679827	0.804410	−0.075809	0.052*	0.5
H1B	0.702894	0.809821	0.048616	0.052*	0.5
H1C	0.691907	0.635769	−0.010649	0.052*	0.5
N2	0.3685 (10)	0.750000	0.5764 (9)	0.035 (3)	
H2C	0.398970	0.864267	0.583555	0.042*	0.5
H2D	0.393159	0.693959	0.521196	0.042*	0.5
H2E	0.404562	0.691774	0.643574	0.042*	0.5
C1	0.8659 (12)	0.750000	0.0129 (13)	0.041 (3)	
H1D	0.898195	0.874116	0.013665	0.049*	0.5
H1E	0.885342	0.682459	−0.048515	0.049*	0.5
C2	0.938 (2)	0.662 (3)	0.127 (2)	0.062 (7)	0.5
H2A	1.034833	0.651088	0.136189	0.075*	0.5
H2B	0.899608	0.541247	0.127654	0.075*	0.5
C3	0.2204 (15)	0.750000	0.5452 (15)	0.066 (5)	
H3A	0.189845	0.874306	0.526024	0.079*	0.5
H3B	0.187034	0.674578	0.476363	0.079*	0.5
C4	0.1613 (18)	0.689 (3)	0.621 (2)	0.066 (9)	0.5
H4A	0.210256	0.749999	0.695318	0.079*	
H4B	0.171565	0.557662	0.627932	0.079*	0.5

### Bis(2-bromoethylammonium) hexabromidostannate(IV) Atomic displacement parameters (Å^2^)

**Table:**

	*U*^11^	*U*^22^	*U*^33^	*U*^12^	*U*^13^	*U*^23^
Sn1	0.0266 (4)	0.0175 (4)	0.0200 (5)	0.000	0.0075 (3)	0.000
Br1	0.0429 (6)	0.0434 (6)	0.0369 (6)	−0.0118 (4)	0.0108 (5)	0.0078 (5)
Br2	0.0354 (7)	0.0432 (8)	0.0214 (8)	0.000	0.0037 (6)	0.000
Br3	0.0500 (6)	0.0354 (6)	0.0426 (7)	−0.0135 (4)	0.0161 (5)	0.0091 (5)
Br4	0.0332 (7)	0.0334 (8)	0.0267 (8)	0.000	0.0029 (6)	0.000
Br5	0.0484 (12)	0.208 (8)	0.0632 (17)	0.029 (2)	−0.0076 (12)	−0.067 (4)
Br6	0.0331 (8)	0.0792 (12)	0.0940 (16)	0.000	0.0242 (9)	0.000
N1	0.032 (6)	0.052 (7)	0.036 (8)	0.000	−0.002 (6)	0.000
N2	0.033 (6)	0.039 (7)	0.032 (7)	0.000	0.010 (5)	0.000
C1	0.029 (7)	0.049 (9)	0.039 (10)	0.000	0.005 (7)	0.000
C2	0.042 (12)	0.063 (15)	0.09 (2)	−0.008 (10)	0.035 (13)	−0.012 (14)
C3	0.034 (9)	0.121 (15)	0.042 (11)	0.000	0.013 (8)	0.000
C4	0.028 (10)	0.06 (2)	0.10 (2)	0.009 (8)	0.008 (12)	0.022 (13)

### Bis(2-bromoethylammonium) hexabromidostannate(IV) Geometric parameters (Å, º)

**Table:**

Sn1—Br1	2.5828 (10)	N2—C3	1.464 (16)
Sn1—Br1^i^	2.5828 (10)	C1—H1D	0.9800
Sn1—Br2	2.6135 (16)	C1—H1D^i^	0.9800
Sn1—Br3^i^	2.5662 (10)	C1—H1E^i^	0.9800
Sn1—Br3	2.5662 (10)	C1—H1E	0.9800
Sn1—Br4	2.6127 (16)	C1—C2^i^	1.51 (3)
Br5—Br5^i^	0.650 (15)	C1—C2	1.51 (3)
Br5—C2	1.88 (2)	C2—H2A	0.9799
Br5—H2B^i^	1.92 (2)	C2—H2B	0.9800
Br6—C4^i^	1.940 (17)	C3—H3A^i^	0.9800
Br6—C4	1.940 (17)	C3—H3A	0.9800
N1—H1A	0.9000	C3—H3B^i^	0.9800
N1—H1B	0.9000	C3—H3B	0.9800
N1—H1C	0.9000	C3—C4	1.35 (2)
N1—C1	1.439 (14)	C3—C4^i^	1.35 (2)
N2—H2C	0.9000	C4—H4A	0.9975
N2—H2D	0.9000	C4—H4B	0.9800
N2—H2E	0.9000		
			
Br1—Sn1—Br1^i^	90.22 (5)	C2^i^—C1—H1E	140.6
Br1^i^—Sn1—Br2	89.11 (4)	C2^i^—C1—H1E^i^	109.3 (9)
Br1—Sn1—Br2	89.11 (4)	C2^i^—C1—C2	51.3 (17)
Br1—Sn1—Br4	89.85 (4)	Br5^i^—C2—Br5	19.8 (5)
Br1^i^—Sn1—Br4	89.85 (4)	Br5—C2—H1D^i^	150.0 (16)
Br3—Sn1—Br1	178.72 (4)	Br5^i^—C2—H1D^i^	156.4 (16)
Br3^i^—Sn1—Br1^i^	178.72 (4)	Br5^i^—C2—H2A	110.1
Br3^i^—Sn1—Br1	88.97 (3)	Br5—C2—H2A	109.0
Br3—Sn1—Br1^i^	88.97 (3)	Br5^i^—C2—H2B	90.5
Br3^i^—Sn1—Br2	89.89 (4)	Br5—C2—H2B	108.8
Br3—Sn1—Br2	89.89 (4)	C1—C2—Br5	112.2 (13)
Br3—Sn1—Br3^i^	91.82 (5)	C1—C2—Br5^i^	127.3 (14)
Br3^i^—Sn1—Br4	91.14 (4)	C1—C2—H1D^i^	39.5 (7)
Br3—Sn1—Br4	91.14 (4)	C1—C2—H2A	108.8
Br4—Sn1—Br2	178.52 (5)	C1—C2—H2B	108.4
Br5^i^—Br5—C2	58.6 (7)	H2A—C2—H1D^i^	93.5
Br5^i^—Br5—H2B^i^	129.7 (7)	H2A—C2—H2B	109.5
C2—Br5—H2B^i^	72.3 (14)	H2B—C2—H1D^i^	80.4
C4^i^—Br6—C4	27.1 (11)	N2—C3—H3A	107.2
H1A—N1—H1B	109.5	N2—C3—H3A^i^	107.22 (13)
H1A—N1—H1C	109.5	N2—C3—H3B	106.1
H1B—N1—H1C	109.5	N2—C3—H3B^i^	106.1 (6)
C1—N1—H1A	109.5	H3A—C3—H3A^i^	140.7
C1—N1—H1B	109.5	H3A^i^—C3—H3B^i^	109.5
C1—N1—H1C	109.5	H3A—C3—H3B^i^	42.0
H2C—N2—H2D	109.5	H3A—C3—H3B	109.5
H2C—N2—H2E	109.5	H3B—C3—H3A^i^	42.0
H2D—N2—H2E	109.5	H3B—C3—H3B^i^	69.7
C3—N2—H2C	109.5	C4^i^—C3—N2	119.6 (15)
C3—N2—H2D	109.5	C4—C3—N2	119.6 (15)
C3—N2—H2E	109.5	C4^i^—C3—H3A	70.4
N1—C1—H1D^i^	109.66 (4)	C4^i^—C3—H3A^i^	107.4 (8)
N1—C1—H1D	109.7	C4—C3—H3A	107.4
N1—C1—H1E^i^	108.8 (6)	C4—C3—H3A^i^	70.4 (10)
N1—C1—H1E	108.8	C4—C3—H3B^i^	132.5 (10)
N1—C1—C2	110.2 (12)	C4^i^—C3—H3B	132.5
N1—C1—C2^i^	110.2 (12)	C4^i^—C3—H3B^i^	106.9 (11)
H1D—C1—H1D^i^	140.2	C4—C3—H3B	106.9
H1D—C1—H1E	109.5	C4^i^—C3—C4	39.3 (17)
H1D^i^—C1—H1E^i^	109.5	Br6—C4—H3A^i^	111.0 (15)
H1D—C1—H1E^i^	51.0	Br6—C4—H4A	107.2
H1E—C1—H1D^i^	51.0	Br6—C4—H4B	109.1
H1E—C1—H1E^i^	61.6	C3—C4—Br6	114.3 (15)
C2^i^—C1—H1D^i^	109.4 (9)	C3—C4—H3A^i^	42.0 (8)
C2—C1—H1D	109.4	C3—C4—H4A	105.4
C2—C1—H1D^i^	61.2 (9)	C3—C4—H4B	109.3
C2^i^—C1—H1D	61.2	H4A—C4—H3A^i^	138.0
C2—C1—H1E	109.3	H4A—C4—H4B	111.5
C2—C1—H1E^i^	140.6 (9)	H4B—C4—H3A^i^	71.7
			
Br5^i^—Br5—C2—C1	143.0 (15)	C2^i^—C1—C2—Br5^i^	49.6 (18)
N1—C1—C2—Br5	−65.4 (13)	C2^i^—C1—C2—Br5	34.8 (13)
N1—C1—C2—Br5^i^	−50.6 (18)	C4^i^—C3—C4—Br6	64.3 (14)
N2—C3—C4—Br6	165.9 (7)		

Symmetry code: (i) *x*, −*y*+3/2, *z*.

### Bis(2-bromoethylammonium) hexabromidostannate(IV) Hydrogen-bond geometry (Å, º)

**Table:**

*D*—H···*A*	*D*—H	H···*A*	*D*···*A*	*D*—H···*A*
N1—H1*A*···Br1^ii^	0.90	2.79	3.481 (9)	134
N1—H1*A*···Br3^iii^	0.90	3.54	3.864 (11)	105
N1—H1*A*···Br3^iv^	0.90	3.08	3.864 (11)	147
N1—H1*A*···Br4^iv^	0.90	3.42	3.7320 (11)	103
N1—H1*B*···Br1	0.90	2.73	3.587 (10)	160
N1—H1*C*···Br1^i^	0.90	3.05	3.587 (10)	121
N1—H1*C*···Br1^v^	0.90	2.87	3.481 (9)	126
N1—H1*C*···Br4^v^	0.90	2.94	3.7320 (11)	147
N2—H2*C*···Br1^vi^	0.90	3.18	3.586 (9)	110
N2—H2*C*···Br2^vii^	0.90	2.87	3.7282 (9)	161
N2—H2*D*···Br2	0.90	2.78	3.462 (10)	133
N2—H2*D*···Br2^viii^	0.90	3.35	3.7282 (9)	108
N2—H2*D*···Br3^i^	0.90	3.55	3.861 (10)	103
N2—H2*D*···Br3	0.90	3.09	3.861 (10)	145
N2—H2*E*···Br1^viii^	0.90	2.71	3.586 (9)	164
C1—H1*D*···Br4^iv^	0.98	3.13	3.872 (3)	133
C1—H1*E*···Br3^iii^	0.98	3.20	3.971 (13)	137
C1—H1*E*···Br4^iii^	0.98	3.56	3.872 (3)	101
C2—H2*A*···Br3^ix^	0.98	2.91	3.73 (2)	142
C2—H2*A*···Br4^ix^	0.98	3.10	3.92 (2)	142
C3—H3*A*···Br3^i^	0.98	3.54	3.934 (14)	107
C3—H3*A*···Br6^x^	0.98	3.31	4.268 (8)	167
C3—H3*B*···Br3	0.98	3.13	3.934 (14)	140
C3—H3*B*···Br6^xi^	0.98	3.52	4.268 (8)	135
C4—H4*B*···Br5^viii^	0.98	2.79	3.54 (2)	134
C4—H4*B*···Br6^xii^	0.98	3.42	4.08 (2)	126

Symmetry codes: (i) *x*, −*y*+3/2, *z*; (ii) −*x*+1, −*y*+2, −*z*; (iii) −*x*+1, −*y*+1, −*z*; (iv) −*x*+1, *y*+1/2, −*z*; (v) −*x*+1, *y*−1/2, −*z*; (vi) −*x*+1, −*y*+2, −*z*+1; (vii) −*x*+1, *y*+1/2, −*z*+1; (viii) −*x*+1, *y*−1/2, −*z*+1; (ix) *x*+1, *y*, *z*; (x) −*x*, *y*+1/2, −*z*+1; (xi) −*x*, −*y*+1, −*z*+1; (xii) −*x*, *y*−1/2, −*z*+1.
